# Maternal and fetal outcomes in patients with congenital heart disease associated with pulmonary arterial hypertension in high-altitude areas: a retrospective cohort study

**DOI:** 10.1186/s12884-026-09340-6

**Published:** 2026-05-28

**Authors:** Xiang-Ting Lu, Xue-Rong Chen, Qing Jin, Yun-Yan Xu, Pei-Yun Jin, Yi-Bing Lu, Hai-Long Dai

**Affiliations:** 1 Department of Cardiology, Clinical Medicine Center, Key Laboratory for Cardiovascular Disease of Yunnan Province, Yan’an Affiliated Hospital of Kunming Medical University, 245 of east Renming road, Kunming, 650051 China; 2 Department of Obstetrics, Yan’an Affiliated Hospital of Kunming Medical University, Kunming, 650051 China

**Keywords:** Pregnancy, Congenital heart disease, Pulmonary arterial hypertension, Maternal and fetal outcomes, High-altitude areas

## Abstract

**Objectives:**

Pulmonary arterial hypertension associated with congenital heart disease (CHD-PAH) increases maternal and fetal risk. This study aimed to analyze maternal and fetal outcomes in patients with CHD-PAH in high-altitude areas.

**Methods:**

The pregnant women with CHD were analyzed retrospectively from 2012 to 2022. Pulmonary artery systolic pressure (PASP) was estimated by echocardiography. Patients were categorized into four groups: no PAH (PASP < 40 mmHg), mild (40 mmHg≤PASP < 50 mmHg), moderate (50 mmHg≤PASP < 70 mmHg) and severe PAH (PASP ≥ 70 mmHg).

**Results:**

429 pregnant women were included in this study, 187 with no PAH, 102 with mild PAH, 79 with moderate PAH, 61 with severe PAH. The severe PAH group had the highest proportions of NYHA functional classes III/IV, SpO_2_<90%, and elevated NT-proBNP levels, but a lower rate of regular prenatal checkups compared with the no PAH and mild PAH groups (*P* < 0.05). The severe PAH group showed significantly higher rates of low birth weight, preterm birth, and neonatal asphyxia (*P* < 0.001). Independent risk factors for maternal and infant complications included NYHA functional classes III-IV (OR = 6.342, 95% CI: 2.28–17.894), NT-proBNP ≥ 250 ng/L (OR = 3.148, 95% CI: 1.121–8.840), moderate PAH (OR = 6.263, 95% CI: 1.657–23.669), and severe PAH (OR = 7.125, 95% CI: 1.792–28.334), whereas higher BMI was a protective factor (OR = 0.802, 95% CI: 0.712–0.904).

**Conclusions:**

The level of PASP, NT-proBNP, and NYHA function class, BMI may be useful in risk stratification in pregnancy with CHD-PAH in high-altitude areas.

## Introduction

It is well known that patients with PAH are poorly adapted to the significant hemodynamic changes caused by pregnancy [1], making them prone to cardiac function deterioration during pregnancy, delivery, and the postpartum period [2]. Pregnant patients with PAH may lead to fetal hypoxia, severely affecting embryonic and fetal development, and may lead to intrauterine growth restriction, fetal distress, neonatal asphyxia, or even death [3, 4]. Therefore, both guidelines and expert consensus recommend that women with PAH should avoid pregnancy [5, 6]. In the event of pregnancy, immediate termination is usually advised [7, 8]. However, real-world data showed that the pregnancy rate among women with PAH is increasing [9, 10] with some patients presenting in the mid-to-late stages of pregnancy at their first clinical visit. Studies have reported that approximately 25% of patients are diagnosed with PAH for the first time during pregnancy [11].

CHD-PAH accounts for approximately 65% of PAH cases during pregnancy, which is the most common etiology of PAH in pregnant women [12, 13]. Some studies showed that the prevalence of CHD is significantly higher in high-altitude areas, approximately 7.21‰, compared to lowland areas [14–16]. Yunnan province is located in the Yunnan-Guizhou Plateau, the average altitude is around 2000 m, the prevalence of CHD is around 6.94%, higher than in other regions of China [17]. Furthermore, CHD patients who living in high-altitude areas are more prone to developing PAH, studies showed that 5% to 10% of adult CHD patients in general developed PAH [6, 18–20], whereas 58.4% of CHD patients in high-altitude areas developed PAH [21].

Different subtypes of PAH, severity of PAH, and medical level lead to different outcomes for maternal and infants [22]. In previous studies, the pregnancy mortality rate of CHD-PAH patients was 30% to 56% [23]. However, in recent decades, significant advances in the understanding of PAH pathogenesis, PAH targeted therapies, and improved intensive care management have contributed to better pregnancy outcomes for patients with PAH [24, 25]. Zhang et al. found that maternal and fetal outcomes in pregnant women with CHD-PAH depended on the severity of PAH, and mild PAH may not be an absolute contraindication to pregnancy in CHD patients [26]. Nevertheless, the substantial physiological changes that occur during pregnancy remain a major challenge for patients with CHD-PAH, especially in those with Eisenmenger syndrome [22, 27]. Currently, data on pregnant women with CHD-PAH in high-altitude areas remain limited. This study retrospectively analyzed the maternal and fetal outcomes and long-term prognosis of pregnant women with CHD-PAH in Yunnan province, which is located in high-altitude areas.

## Methods

### Study population, data sources, and measures

**Fig. 1:**
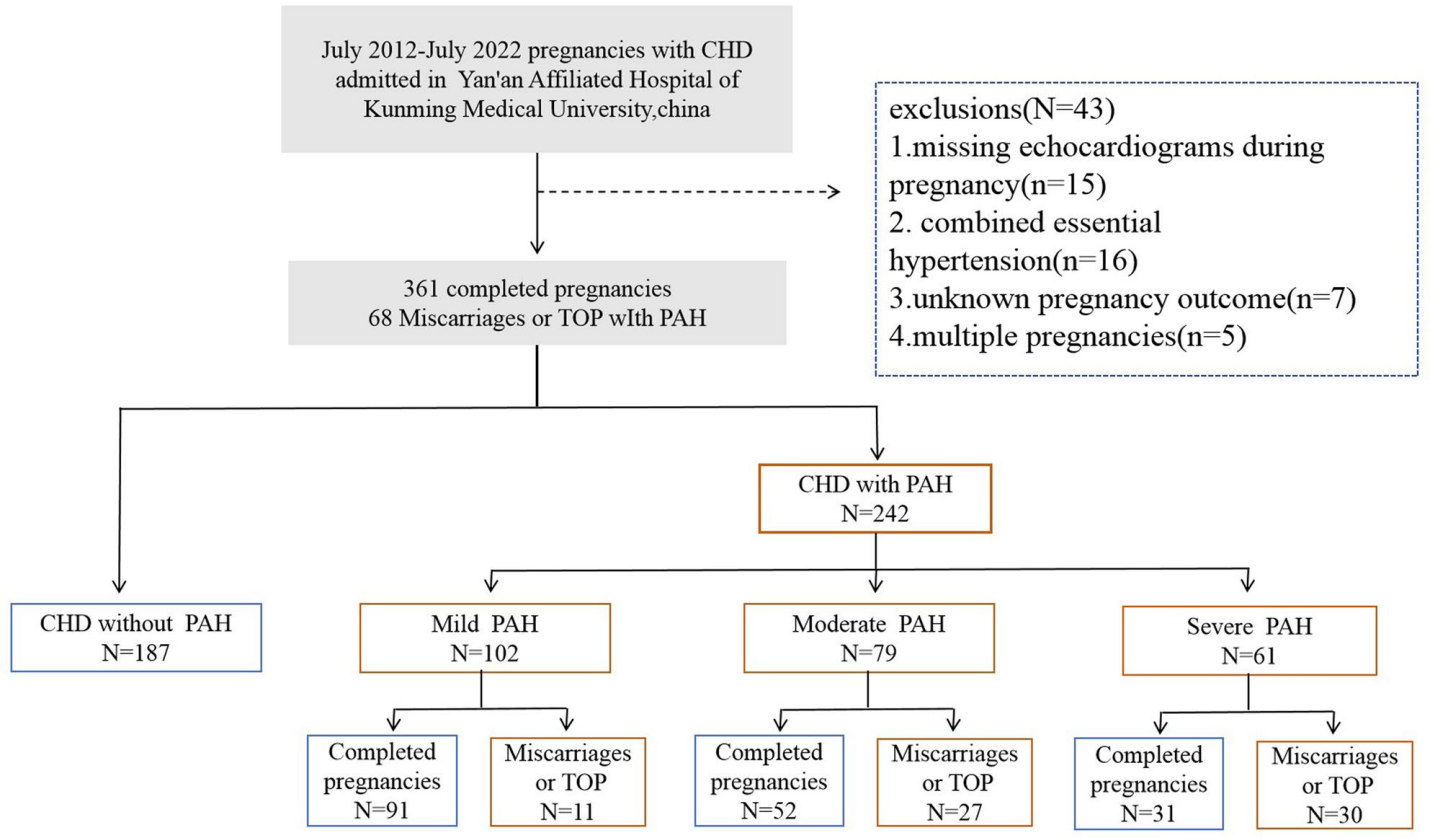
Flow chart of inclusion. CHD: Congenital heart disease; PAH: Pulmonary artery hypertension; TOP: Termination of pregnancy

Pregnant women with CHD who were hospitalized between July 2012 and July 2022 were included in this study (Fig. 1). Inclusion criteria: (1) Pregnant women diagnosed with CHD by echocardiography; (2) Singleton pregnancy; (3) Definite pregnancy outcomes. Exclusion criteria: (1) PAH caused by other etiologies; (2) Hospitalized but did not deliver, with unknown pregnancy outcomes; (3) patients who had incomplete medical records; (4) Pregnancies complicated by a scarred uterus or multiple gestations.

According to the pulmonary artery systolic pressure (PASP) measured by echocardiography at admission, patients were divided into four groups [28, 29]: no PAH group (PASP < 40 mmHg); mild PAH group (40 mmHg ≤ PASP < 50 mmHg); moderate PAH group (50 mmHg ≤ PASP < 70 mmHg); severe PAH group (PASP ≥ 70 mmHg).

Cardiac complications included worsening heart failure, heart failure requiring treatment, and death. Obstetric events included preterm birth, postpartum hemorrhage, gestational hypertension, gestational diabetes, and preeclampsia. Neonatal outcomes included low birth weight, very low birth weight, neonatal asphyxia, and neonatal death. Maternal and fetal complications were defined as the occurrence of any cardiac complication, obstetric event, offspring event, or miscarriage.

Clinical data of enrolled patients were collected from the electronic medical record system. Baseline characteristics included: age, gravidity and parity, body mass index (BMI), gestational age at admission, whether prenatal examinations were conducted, length of hospital stay, timing of CHD diagnosis. Main symptoms and medical history. Relevant laboratory and auxiliary examinations: N-terminal pro-B-type natriuretic peptide (NT-proBNP), albumin (ALB), total bilirubin (TBIL), pulse oxygen saturation (SpO_2_), electrocardiogram (ECG), echocardiography results for both mother and fetus; Maternal and fetal outcomes: gestational age at delivery, mode of delivery, type of anesthesia, neonatal birth weight, and maternal and neonatal complications.

Follow-up was conducted via telephone to assess maternal survival outcomes. The follow-up deadline was February 2024.

### Statistical analysis

Measurement data with normal distribution were expressed as mean ± standard deviation; comparisons among groups were conducted using analysis of variance (ANOVA), and pairwise comparisons were performed using the Bonferroni correction. Measurement data with non-normal distribution were expressed as median (Q1, Q3); group comparisons were conducted using the Kruskal-Wallis rank-sum test. Categorical data were expressed as number (percentage), and comparisons among groups were conducted using the Chi-square test or Fisher’s exact test. Binary logistic regression was used to analyze independent risk factors for maternal and neonatal complications. Variables with *P* < 0.05 in univariate analysis were included in the multivariate logistic regression model; *P* < 0.05 indicated statistical significance.

## Results

### General clinical data

A total of 429 eligible pregnant women with CHD were included, with a mean age of 27 ± 5 years. Among them, 187 (43.6%) had no PAH, 102 (23.8%) had mild PAH, 79 (18.4%) had moderate PAH, and 61 (14.2%) had severe PAH. The most common CHD subtype was atrial septal defect (54.3%), followed by ventricular septal defect (23.8%), while patent ductus arteriosus, compound malformations, Tetralogy of Fallot, and complete atrioventricular septal defect accounted for smaller proportions. Overall, 167 patients (38.9%) had undergone defect repair before pregnancy.

CHD patients were categorized into four groups: no PAH (43.6%), mild PAH (23.8%), moderate PAH (18.4%), and severe PAH (14.2%). Baseline age, ALB < 30 g/L, and TBIL levels were no differences among groups. Compared with the other groups, patients with severe PAH showed worse clinical status, including higher proportions of NYHA functional class III/IV, SpO₂ <90%, NT-proBNP ≥ 250 ng/L, and reduced LVEF, as well as lower rates of regular prenatal care (all *P* < 0.05). Moderate and severe PAH groups also had lower BMI, larger right ventricular diameter, and higher rates of arrhythmia. In contrast, patients without PAH more frequently underwent preconception diagnosis and defect repair before pregnancy and had the lowest incidence of pericardial effusion (Table 1).

**Table 1:** Baseline characteristics

	No PAH (*N* = 187)	Mild PAH (*N* = 102)	Moderate PAH (*N* = 79)	Severe PAH (*N* = 61)	*P* value
Age, y, mean ± SD	27.2 ± 4.5	28.5 ± 5.4	27.2 ± 5.4	26.5 ± 5.8	0.127
BMI, kg/m^2^, mean ± SD	26.1 ± 3.9^bc^	25.9 ± 3.3^bc^	24.2 ± 3.7^c^	21.6 ± 3.7	< 0.001
Advanced maternal age, n (%)	9(4.8%)^a^	15(14.7%)	9(11.4%)	6(9.8%)	0.035
Primipara, n (%)	134(71.7%)^abc^	51(50.0%)^c^	33(41.8%)^c^	12(19.7%)	< 0.001
Regular prenatal checkups, n (%)	125(66.8%)^c^	65(63.7%)^c^	50(63.3%)	25(41.0%)	0.004
Preconception diagnosis, n (%)	146(78.1%)^abc^	19(18.6%)	13(16.5%)	20(32.8%)	< 0.001
Arrhythmia requiring treatment, n (%)	81(43.3%)	39(38.2%)^bc^	47(59.5%)	37(60.7%)	0.003
Unrepaired CHD, n (%)	52(27.8%)^abc^	91(89.2%)	69(87.3%)	50(82.0%)	< 0.001
NT-proBNP, ng/L, median (IQR)	56 (30,96)^c^	39 (17,90)^c^	59 (37,147)^c^	211(53,571)	< 0.001
NT-proBNP, n (%)					
≥ 250ng/L	9(4.8%)^c^	5(4.9%)^c^	4(5.1%)^c^	20(32.8%)	< 0.001
SpO_2_<90%, n (%)	3(1.6%)^c^	0(0.0%)^c^	0(0.0%)^c^	19(31.7%)	< 0.001
ALB<30 g/L, n (%)	28(15.2%)	11(10.8%)	8(10.1%)	13(21.7%)	0.175
NYHA, n (%)					
I-II	174(93.0%)^bc^	95(93.3%)^bc^	62(78.5%)	35(57.4%)	
III-IV	13(7.0%)^bc^	7(6.7%)^bc^	17(21.5%)	26(42.6%)	< 0.001
LVEF < 50%, n (%)	3(1.6%)	0(0.0%)	1(1.3%)	5(8.2%)	-
LVEDD, mm, median (IQR)	46(44, 50)^ab^	44(40, 50)	43(39, 48)	45(38, 54)	0.001
RVD, mm, median (IQR)	20(18, 23)^abc^	27(22, 30)^b^	33(23, 37)^c^	26(23, 30)	< 0.001
PASP, mmHg, median (IQR)	-	40(40, 45)	55(50, 60)	90(75, 100)	< 0.001
Pericardial effusion, n (%)	4(2.1%)^abc^	14(13.7%)	8(10.1%)	10(16.4%)	< 0.001

Compared with the mild PAH group,^a^*P*<0.01; Compared with the moderate PAH group, ^b^*P*<0.01; Compared with the severe PAH group, ^c^*P*<0.01

*PAH *Pulmonary artery hypertension, *BMI *Body mass index, *CHD *Congenital heart disease, *NT-proBNP *N-terminal pro-brain natriuretic peptide, *SpO*_2_ Saturation of peripheral oxygen, *ALB *Albumin, *TBIL *Total bilirubin, *NYHA *New York Heart Association, *LVEF *Left ventricular ejection fraction, *PASP *Pulmonary artery systolic pressure *LVEDD* Left ventricular end-diastolic diameter, *RVD* Right ventricular dimension

### Main symptoms and signs

The most common symptoms were dyspnea (9.8%), chest tightness (7.0%), and palpitations (6.5%), while edema, cyanosis, and clubbing were the most common physical signs. Severe PAH patients were associated with significantly higher frequencies of dyspnea, chest tightness, cough, and cyanosis compared with no PAH or mild PAH groups. Edema was more common in moderate and severe PAH patients, whereas palpitations were more frequent in the moderate PAH patients (all *P* < 0.05) (Table 2).

**Table 2:** Clinical symptoms

Clinical symptoms	No PAH (*N* = 187)	Mild PAH (*N* = 102)	Moderate PAH (*N* = 79)	Severe PAH (*N* = 61)	*P* Value
Symptoms					
Dyspnea	0(0.0%)	2(2.0%)	3(3.8%)	3(4.9%)	-
Gasp	8(4.3%)^c^	6(5.9%)^c^	9(11.4%)^c^	19(31.1%)	<0.001
Chest tightness	4(2.1%)^ac^	10(9.8%)	7(8.9%)	9(14.8%)	0.003
Palpitation	6(3.2%)^b^	5(4.9%)	11(13.9%)	6(9.8%)	0.007
Cough	0(0.0%)^c^	0(0.0%)	1(1.3%)	4(6.6%)	-
Signs					
Cyanosis	2(1.1%)	0(0.0%)	2(2.5%)	14(23.0%)	-
Clubbing	0(0.0%)	6(5.9%)	0(0.0%)	7(11.5%)	-
Edema	2(1.1%)^bc^	6(5.9%)	9(11.4%)	6(9.8%)	0.002

Compared with the mild PAH group, ^a^*P*<0.01; Compared with the moderate PAH group, ^b^*P*<0.01; Compared with the severe PAH group, ^c^*P*<0.01

### Maternal outcomes

223/429 patients (52.0%) received multidisciplinary team consultation, and 68/429 (15.9%) patients with uncompleted pregnancies (miscarriage or termination of pregnancy) at a mean gestational age of 15.6 ± 7.0 weeks. The rate of uncompleted pregnancies increased progressively with PAH severity, ranging from 0% in the no PAH group to 49.2% in the severe PAH group (*P* < 0.001). Compared with no PAH and mild PAH groups, severe PAH was associated with longer hospital stay, shorter gestational duration, and higher rates of general anesthesia (all *P* < 0.05) (Table 3).

**Table 3:** Clinical outcomes and management in patients with No PAH, Mild PAH, Moderate PAH and Severe PAH

	No PAH (*N* = 187)	Mild PAH (*N* = 102)	Moderate PAH (*N* = 79)	Severe PAH (*N* = 61)	*P*
Maternal cardiac events, *n* (%)					
Death	0(0.0%)	0(0.0%)	0(0.0%)	4(6.6%)	-
Worsening heart failure	8(4.3%)^c^	5(4.9%)^c^	7(8.9%)^c^	20(32.8%)	< 0.001
Arrhythmia requiring treatment	2(1.1%)^b^	2(2.0%)	7(8.9%)	3(4.9%)	0.008
Obstetric events, n (%)					
Preterm delivery (< 37 wk)	10(5.3%)^c^	5(5.5%)^c^	8(15.4%)	12(38.7%)	< 0.001
Postpartum hemorrhage	1(0.5%)	0(0.0%)	0(0.0%)	0(0.0%)	1.000
Pregnancy induced hypertension	7(3.7%)	6(5.9%)	2(2.5%)	3(4.9%)	0.697
Gestational diabetes	10(5.3%)	2(2.0%)	2(2.5%)	0(0.0%)	0.198
Preeclampsia	0(0.0%)	2(2.0%)	1(1.3%)	1(1.6%)	-
Offspring events, n (%)					
Offspring death	0(0.0%)	0(0.0%)	1(1.9%)	1(3.2%)	0.052
Low birth weight	13(7%)^c^	6(6.6%)^c^	8(15.4%)^c^	13(41.9%)	< 0.001
Very low birth weight	2(1.1%)^c^	0(0.0%)^c^	0(0.0%)	3(9.7%)	0.012
Neonatal asphyxia	1(0.5%)^c^	0(0.0%)^c^	3(5.8%)	3(9.7%)	< 0.001
Offspring CHD	7(3.7%)^bc^	8(8.8%)^c^	10(19.2%)^c^	16(51.6%)	< 0.001
Management					
Length of hospital stay, d, median (IQR)	5(4, 6)^bc^	5(4, 6)^c^	5(4, 7)^c^	7(4, 9)	< 0.001
MDT, n (%)	6(3.2%)^abc^	88(86.3%)	70(88.6%)	59(96.7%)	< 0.001
pregnancy outcomes, n (%)					
Miscarriages or termination of pregnancy	0(0.0%)^abc^	11(10.8%)^bc^	27(34.2%)	30(49.2%)	< 0.001
completed pregnancy	187(100%)^abc^	91(89.2%)^bc^	52(65.8%)	31(50.8%)	
Time of miscarriage, wk, mean ± SD	-	15.1 ± 7.8	16.8 ± 6.9	14.7 ± 6.8	0.519
Gestational week at delivery, median (IQR)	38(38, 39)^c^	38(38, 39)^c^	38(37, 39)	37(35, 38)	0.004
Mode of delivery n (%)					
Caesarean section	165(88.2%)	88(96.7%)	50(96.2%)	30(96.8%)	0.033
Vaginal	22(11.8%)	3(3.3%)	2(3.8%)	1(3.2%)	
Anesthesia n (%)					
Regional anesthesia	157(84%)^bc^	89(87.3%)^bc^	50(63.3%)	31(50.8%)	< 0.001
General anesthesia	9(4.8%)^c^	1(1.0%)^c^	3(3.8%)^c^	12(19.7%)	
Others	21(11.2%)^bc^	12(11.8%)^bc^	26(32.9%)	18(29.5%)	
Drug therapy, n (%)					
Monotherapy	-	0(0.0%)^c^	2(2.5%)^c^	14(23.0%)	< 0.001
Combination therapy	-	0(0.0%)	0(0.0%)	1(1.6%)	

Compared with the mild PAH group, ^a^*P*<0.01; Compared with the moderate PAH group, ^b^*P*<0.01; Compared with the severe PAH group, ^c^*P*<0.01

Cardiac and obstetric complications increased with PAH severity. Overall, 361/429 patients (84.1%) with completed pregnancies, 35/361 (9.7%) pregnant women had preterm delivery, and 326/361 (90.3%) pregnant women delivered at full term. Notably, two maternal deaths occurred in the uncompleted pregnancies group and two in the preterm delivery group. Patients with uncompleted pregnancies had higher proportions of severe PAH, NYHA functional class III-IV, and worsening heart failure compared with those delivering at full term (all *P* < 0.05) (Table 4). Overall, worsening heart failure occurred in 40/429 patients (9.3%), arrhythmias requiring treatment occurred in 14/429 patients (3.3%), and maternal death occurred in 4/429 patients (0.9%). Maternal deaths and worsening heart failure were most common in the severe PAH group, whereas arrhythmias requiring treatment occurred more frequently in the moderate PAH group. No significant differences were observed between the no PAH and mild PAH groups (Table 3).

**Table 4:** Clinical characteristics of patients with uncompleted pregnancies, preterm delivery, and full-term delivery

	Uncompleted pregnancies(*n* = 68)	Preterm delivery (*n* = 35)	Full-term delivery (*n* = 326)	*P* value
PAH, n (%)				
No PAH	0(0.0%)^ab^	10(28.6%)^b^	177(54.3%)	< 0.001
Mild PAH	11(16.2%)	5(14.3%)	86(26.4)	
Moderate PAH	27(39.7%)^b^	8(22.9%)	44(13.5%)	
Severe PAH	30(44.1%)^b^	12(34.3%)^b^	19(5.8%)	
Maternal cardiac events, n (%)				
NYHA III-IV	22(32.4%)^b^	15(42.9%)^b^	26(8.0%)	< 0.001
Arrhythmia requiring treatment	3(4.4%)	1(2.9%)	10(3.1%)	0.843
Worsening heart failure	11(16.2%)^b^	10(28.6%)^b^	19(5.8%)	< 0.001
Death	2(2.9%)	2(5.7%)	0(0.0%)	-

Compared with the preterm delivery group,^a^*P*<0.01; Compared with the full-term delivery group, ^b^*P*<0.01

*PAH *pulmonary artery hypertension, *NYHA *New York Heart Association

Among obstetric complications, preterm delivery was the most common event, followed by gestational hypertension and gestational diabetes. The rate of preterm delivery was significantly higher in severe PAH patients compared with no PAH and mild PAH groups (*P* < 0.05) (Table 3).

### Fetal outcomes

Among the 361 completed pregnancies, low birth weight occurred in 40 neonates (11.1%), very low birth weight in 5 (1.4%), neonatal asphyxia in 7 (2.0%), and neonatal death in 2 (0.6%). Neonatal adverse outcomes increased with PAH severity and were most frequent in the severe PAH group, including low birth weight, very low birth weight, and neonatal asphyxia (all *P* < 0.05) (Table 3).

**Fig. 2:**
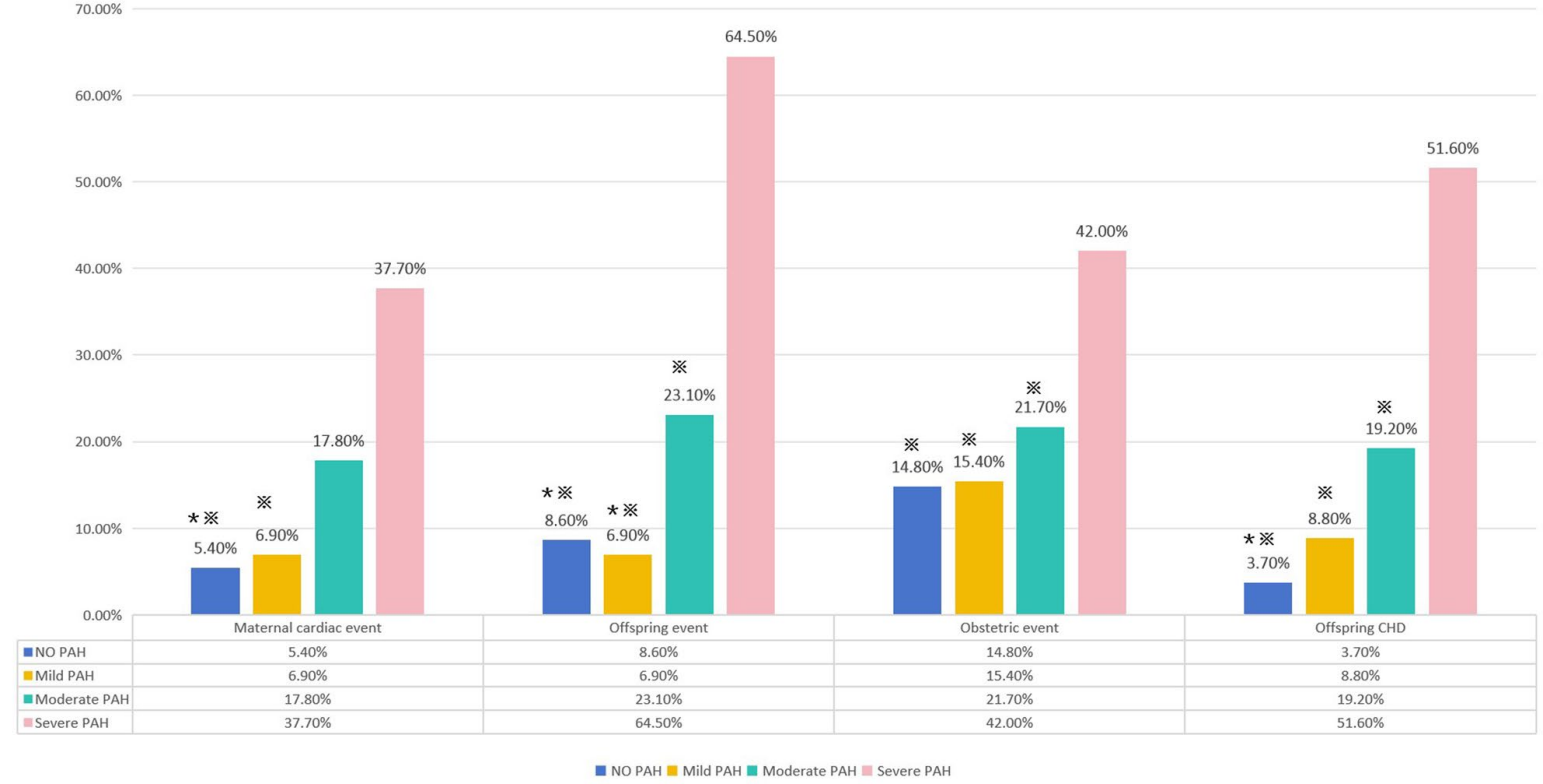
Adverse events in CHD patients with PAH and without PAH. Compared with severe PAH, ※*P*<0.01; Compared with moderate PAH, *P<0.01;CHD: Congenital heart disease; PAH, Pulmonary artery hypertension

The incidence of CHD in offspring also increased with PAH severity and was highest in the severe PAH group (51.6%). Compared with no PAH or mild PAH, moderate and severe PAH were associated with significantly higher rates of maternal cardiac events, adverse neonatal outcomes, and CHD in offspring (Fig. 2).

### Clinical characteristics of patients with uncompleted and completed pregnancies

361/429 patients (84.1%) with completed pregnancies, while 85/361 (23.5%) patients with completed pregnancies with adverse events. Patients with uncompleted pregnancies had the lowest BMI, prenatal diagnosis rate, and proportion of repaired CHD, while exhibiting the largest right ventricular diameter. Compared with patients who completed pregnancy without adverse events, those with uncompleted pregnancies or completed pregnancy with adverse events had higher NT-proBNP levels, PASP, rates of SpO₂ <90%, ALB < 30 g/L, and NYHA functional class III-IV (all *P* < 0.05). The prevalence of severe PAH was highest in uncompleted pregnancies (Table 5).

**Table 5:** Clinical characteristics of patients with uncompleted pregnancies and completed pregnancy

	Uncompleted pregnancies(*n* = 68)	Completed pregnancies with adverse events (*N* = 85)	Completed pregnancies without adverse events (*N* = 276)	*P* value
Age, y, mean ± SD	26.7 ± 5.3	28.0 ± 5.4	27.4 ± 5.0	0.288
BMI, kg/m^2^, mean ± SD	20.7 ± 2.9^ab^	24.8 ± 4.6^b^	26.3 ± 3.2	< 0.001
Advanced maternal age, n (%)	6(8.8%)	11(12.9%)	21(7.6%)	0.318
Primipara, n (%)	26(38.2%)^b^	39(45.9%)	165(59.8%)	0.002
Gestational week at delivery, w, mean ± SD	15.6 ± 7.0 ^ab^	36.8 ± 2.3 ^b^	38.3 ± 1.0	< 0.001
Regular prenatal checkups, n (%)	43(63.2%)	41(48.2%)	173(62.7%)	0.049
Preconception diagnosis, n (%)	17(25.0%)^ab^	40(47.1%)	141(51.1%)	0.001
Unrepaired CHD, n (%)	59(86.8%)^ab^	54(63.5%)	149(54.0%)	< 0.001
MDT	62(91.2%)^ab^	47(55.3%)	114(41.3%)	< 0.001
NT-proBNP, ng/L, median (IQR)	97.5(41.5, 343.8) ^b^	100.5(31.8, 304.0) ^b^	50.0(25.3, 91.0)	0.001
≥ 250ng/L	11(16.2%) ^b^	16(18.8%) ^b^	11(4.0%)	< 0.001
SpO_2_<90%, n (%)	10(14.7%)^b^	10(11.8%)^b^	0(0.0%)	< 0.001
ALB<30 g/L, n (%)	4(5.9%)^a^	22(25.9%)^b^	33(12.0%)	0.001
NYHA, n (%)				
I-II	46(67.6%)	60(70.6%)	260(94.2%)	< 0.001
III-IV	22(32.4%)^b^	25(29.4%)^b^	16(5.8%)	
LVEF(%)				
<50%	1(1.5%)	3(3.5%)	4(1.4%)	-
LVEDD, %, median (IQR)	41.5(37.0, 49.8)^b^	45.0(40.5, 50.0)	46.0(42.3, 50.0)	< 0.001
RVD	29.6 ± 7.0^ab^	25.5 ± 7.4	24.0 ± 7.0	< 0.001
PASP, mmHg, median (IQR)	60.0(50.0, 91.3)^b^	55.0(45.0, 70.0)^b^	45.0(40.0,50.0)	< 0.001
PAH, n (%)				
No PAH	0(0.0%)^ab^	35(41.2%)	152(55.1%)	< 0.001
Mild PAH	11(16.2%)	14(16.5%)	77(27.9%)	
Moderate PAH	27(39.7%)^b^	18(21.2%)	34(12.3%)	
Severe PAH	30(44.1%)^ab^	18(21.2%)^b^	13(4.7%)	
Pericardial effusion, n (%)	8(11.8%)	8(9.4%)	18(6.5%)	0.305

Compared with the completed pregnancies with adverse events group,^a^*P*<0.01;Compared with the completed pregnancies without adverse events group, ^b^*P*<0.01

*PAH *Pulmonary artery hypertension, *BMI *Body mass index, *CHD *Congenital heart disease; *NT-proBNP *N-terminal pro-brain natriuretic peptide, *SpO*_2_ Saturation of peripheral oxygen, *ALB *Albumin, *TBIL *Total bilirubin, *NYHA *New York Heart Association, *LVEF *Left ventricular ejection fraction, *PASP *Pulmonary artery systolic pressure, *MDT *Multidisciplinary team, *LVEDD *Left Ventricular End-Diastolic Dimension, *RVD *Right ventricle diameter

### Risk factors for maternal and fetal complications

**Fig. 3:**
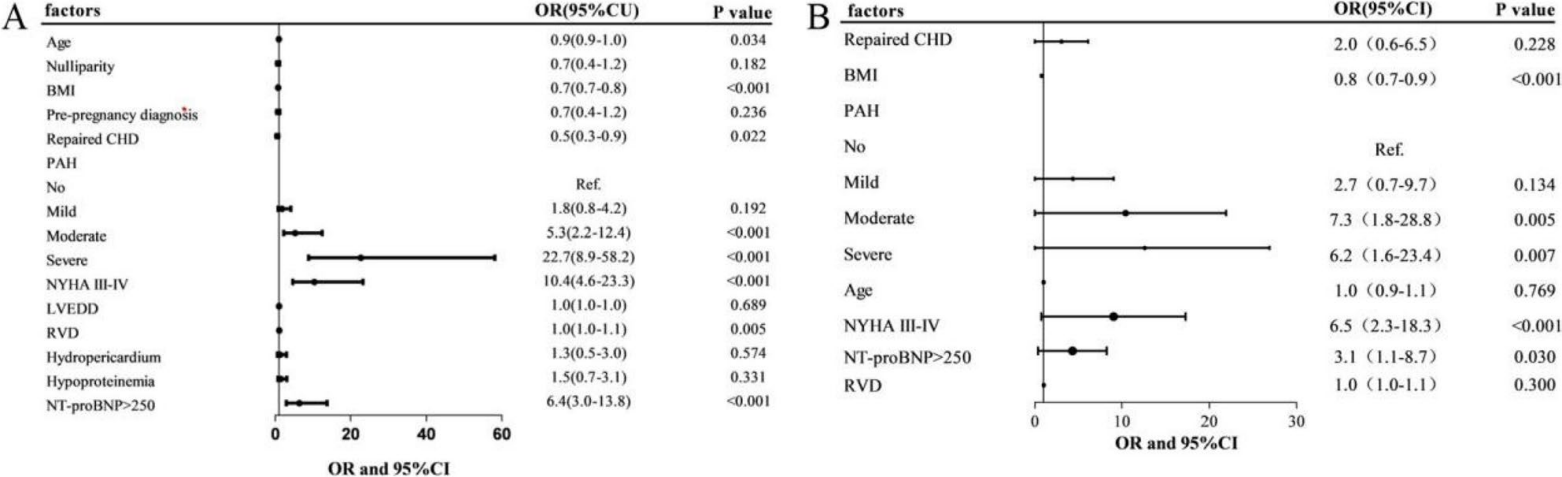
Univariable and multivariable analyses of maternal and offspring event in CHD patients. Univariable (**A**) and multivariable (**B**) analyses were performed to explore the risk factors associated with maternal and offspring complications in patients with CHD. BMI: Body mass index; CHD: Congenital heart disease; NYHA: New York Heart Association; OR: Odds ratio; PAH: Pulmonary artery hypertension; NT-proBNP: N-terminal pro-brain natriuretic peptide; LVEF: Left ventricular ejection fraction; LVEDD: Left ventricular end-diastolic diameter; PASP: Pulmonary artery systolic pressure

Univariable analysis showed that defect repair surgery (OR = 0.5, 95% CI: 0.3–0.9), high BMI (OR = 0.7, 95% CI: 0.7–0.8), and older age (OR = 0.9, 95% CI: 0.9–1.0)were associated with a lower incidence of maternal and infant complications, while moderate PAH (OR = 5.3, 95% CI: 2.2–12.4), severe PAH (OR = 22.7, 95% CI: 8.9–58.2), NYHA functional class III-IV (OR = 10.4, 95% CI: 4.6–23.3), NT-proBNP ≥ 250 ng/L (OR = 6.4, 95% CI: 3.0–13.8), and right ventricular enlargement (OR = 1.0, 95% CI: 1.0–1.1) are associated with a high incidence of complications (Fig. 3A). Multivariate logistic regression analysis revealed that NYHA functional class III-IV (OR = 6.517, 95% CI: 2.321–18.299), NT-proBNP ≥ 250 ng/L (OR = 3.12, 95% CI: 1.116–8.725), Moderate PAH (OR = 6.201, 95% CI: 1.64–23.447), and severe PAH (OR = 7.254, 95% CI: 1.826–28.826) were independent risk factors for maternal and fetal complications, while high BMI (OR = 0.802, 95% CI: 0.712–0.904) was a protective factor (Fig. 3B).

### Follow-up

Among the 425 patients who survived hospitalization, follow-up was completed in 421 patients over a mean duration of 5.8 ± 2.79 years. Four patients were lost to follow-up, and four patients died during the postpartum follow-up period, including two patients with moderate PAH and two with severe PAH.

## Discussion

To our knowledge, this is the largest study on pregnant women with CHD-PAH in high-altitude areas of China. This study included 429 pregnant women with CHD, 361 (84.1%) completed their pregnancies. Firstly, we found that pregnant women with severe PAH had a higher incidence of maternal and fetal complications compared to those with no PAH or mild PAH. Compared to pregnant women with severe PAH, those with moderate PAH had lower incidences of neonatal adverse outcomes, obstetric complications. Pregnant women with no PAH or mild PAH had the lowest rates of these adverse outcomes, with no difference between these two groups. Second, our study showed that moderate to severe PAH, NYHA functional class III-IV, and NT-proBNP ≥ 250 ng/L were associated with a higher incidence of maternal and fetal complications. In contrast, a higher BMI and a history of defect repaired were associated with a lower incidence of maternal and fetal complications. These findings suggest that moderate to severe PAH, NYHA functional class III-IV, and NT-proBNP ≥ 250 ng/L may help stratify the risk for pregnant women with CHD-PAH.

Our study also indicates the potential need for more personalized pregnancy risk assessment strategies for CHD-PAH patients. In our study, the maternal mortality rate in CHD-PAH patients was 0.9%, which is lower than previously reported [23, 24]. Half of the women with severe PAH opted for medically indicated pregnancy termination, previous studies have shown that early termination of pregnancy can reduce mortality in high-risk patients, however, we found that the rate of worsening heart failure remained high. It is worth noting that all four in-hospital deaths occurred in patients with Eisenmenger syndrome, including two who had early pregnancy termination, suggesting that pregnancy should be contraindicated in women with Eisenmenger syndrome, consistent with previous studies [11, 27–30]. In our study, the maternal mortality rate for Eisenmenger syndrome was 28.6%. For these patients, comprehensive multidisciplinary management is particularly important, and early pregnancy termination may help prevent maternal death [31].

In our study, compared to patients without PAH, pregnant women with CHD-PAH had higher rates of death, worsening heart failure, arrhythmias requiring treatment, preterm birth, low birth weight infants, and other adverse events, consistent with previous findings [23, 26]. In our study, some high-risk patients chose to continue their pregnancy for the following reasons: Firstly, some women in rural or remote areas were already in the middle to late stages of pregnancy when seeking medical attention. Secondly, regular prenatal care and multidisciplinary consultations were less frequent in this group. Among the four in-hospital deaths, three patients had never received any prenatal care and were already in critical condition upon admission to our hospital. Studies have shown that early multidisciplinary team (MDT) evaluation, including timely initial prenatal visits, MDT follow-up, and strict prenatal supervision, can significantly improve patient outcomes [26, 32]. Therefore, in order to detect PAH early and promptly refer them to specialized hospitals, we need to improve standardized antenatal management and referral systems in high-altitude areas.

In our study, patients who no adverse maternal and fetal events were predominantly those with no PAH or mild PAH, accounting for approximately 83.0%. These patients have better heart functions and lower NT-proBNP levels. Additionally, they had high BMI, pre-pregnancy diagnosis, and cardiac defect repair rate. This study suggests that pregnancy may not be an absolute contraindication in CHD patients with no or mild PAH, which is consistent with previous studies [26]. Multivariate analysis revealed that moderate/severe PAH, NYHA functional class III/IV, and NT-proBNP ≥ 250 ng/L were independent risk factors for adverse maternal and fetal events. This is consistent with previous findings [33, 34], which demonstrated that high pulmonary artery pressures are associated with increased risk of adverse maternal and fetal outcomes. Pregnant women with mild PAH had significantly better outcomes compared to those with moderate to severe PAH. Our study found that a high BMI was a protective factor against adverse maternal and fetal outcomes, however, does not imply a causal protective effect of obesity. The impact of obesity on PAH remains unclear. An obesity paradox, wherein patients who are obese have lower mortality, has been described in PAH [35–36]. The possible reason for this contradiction may be that in our study, high-risk patients had severe conditions and right heart failure led to systemic congestion, and impaired digestive function, resulting in malnutrition and reduced skeletal muscle mass, potentially causing respiratory hypofunction, further worsening lung function and increasing mortality rates. In addition, malnutrition can also increase respiratory muscle atrophy and infection susceptibility, subsequently impairing lung function. Current research indicates that improving dietary quality during pregnancy is associated with a reduced risk of CHD in offspring [37].Therefore, PAH multidisciplinary teams should include specialists in nutrition and dietetics to establish nutrition intervention strategies.

Interestingly, this study found that the incidence of CHD in offspring of pregnant women with CHD was 11.4%, significantly higher than that reported in CHD patients from non-high-altitude areas [14–16]. Moreover, the incidence of CHD in the offspring of women with severe CHD-PAH was even higher (51.6%), which may be related to severe maternal hypoxia leading to impaired fetal cardiovascular development, as well as potential genetic susceptibility and shared familial risk factors.

### Limitations

The primary strength of this study lies in its relatively large population-based sample and comprehensive collection of clinical, obstetric, and neonatal indicators relevant to pregnant women with CHD-PAH residing in high-altitude areas. This provides valuable evidence from a region where such data remain scarce.

Several limitations should be acknowledged. First, PAH diagnosis was based on echocardiographic estimates rather than right heart catheterization, which may have resulted in over- or underestimation of pulmonary artery pressures, particularly among patients near the diagnostic threshold. Additionally, elevated pulmonary pressures during pregnancy may partly reflect increased cardiac output rather than true pulmonary vascular disease. Second, this was a single-center study, which may limit the generalizability of the findings to broader populations. Third, most patients originated from remote rural areas, contributing to low follow-up rates and incomplete availability of postpartum echocardiographic data. Finally, our research spans a long period and there are some potential selection biases and changes in management practices, including advances in therapeutic drugs, strategies, and improvements in economic living standards, which may have influenced the study outcomes.

## Conclusions

In high-altitude areas, the outcomes of pregnant women with mild CHD-PAH seems better than those in moderate and severe CHD-PAH, similar to CHD without PAH patients. Moderate to severe PAH, NT-proBNP≥250ng/L, and NYHA functional class III-IV are independent risk factors for maternal and fetal adverse events in pregnant women with CHD-PAH, whereas high BMI was a protective factor.

## Data Availability

The datasets used and/or analysed during the current study available from the corresponding author on reasonable request.
